# Melatonin-stimulated MSC-derived exosomes improve diabetic wound healing through regulating macrophage M1 and M2 polarization by targeting the PTEN/AKT pathway

**DOI:** 10.1186/s13287-020-01756-x

**Published:** 2020-06-29

**Authors:** Wei Liu, Muyu Yu, Dong Xie, Longqing Wang, Cheng Ye, Qi Zhu, Fang Liu, Lili Yang

**Affiliations:** 1 Spine Center, Department of Orthopaedics, Shanghai Changzheng Hospital, Second Military Medical University, Shanghai, 200003 China; 2 Department of Endocrinology and Metabolism, Shanghai Diabetes Institute, Shanghai Key Laboratory of Diabetes Mellitus, Shanghai Clinical Center for Diabetes, Shanghai Jiao Tong University Affiliated Sixth People’s Hospital, Shanghai, 200233 China

**Keywords:** Exosome, Mesenchymal stem cell, Melatonin, Macrophage polarization, Diabetic wound, Inflammation

## Abstract

**Background:**

After surgery, wound recovery in diabetic patients may be disrupted due to delayed inflammation, which can lead to undesired consequences, and there is currently a lack of effective measures to address this issue. Mesenchymal stem cell (MSC)-derived exosomes (Exo) have been proven to be appropriate candidates for diabetic wound healing through the anti-inflammatory effects. In this study, we investigated whether melatonin (MT)-pretreated MSCs-derived exosomes (MT-Exo) could exert superior effects on diabetic wound healing, and we attempted to elucidate the underlying mechanism.

**Methods:**

For the evaluation of the anti-inflammatory effect of MT-Exo, in vitro and in vivo studies were performed. For in vitro research, we detected the secreted levels of inflammation-related factors, such as IL-1β, TNF-α and IL-10 via ELISA and the relative gene expression of the IL-1β, TNF-α, IL-10, Arg-1 and iNOS via qRT-PCR and investigated the expression of PTEN, AKT and p-AKT by Western blotting. For in vivo study, we established air pouch model and streptozotocin (STZ)-treated diabetic wound model, and evaluated the effect of MT-Exo by flow cytometry, optical imaging, H&E staining, Masson trichrome staining, immunohistochemical staining, immunofluorescence, and qRT-PCR (α-SMA, collagen I and III).

**Results:**

MT-Exo significantly suppressed the pro-inflammatory factors IL-1β and TNF-α and reduced the relative gene expression of IL-1β, TNF-α and iNOS, while promoting the anti-inflammatory factor IL-10 along with increasing the relative expression of IL-10 and Arg-1, compared with that of the PBS, LPS and the Exo groups in vitro. This effect was mediated by the increased ratio of M2 polarization to M1 polarization through upregulating the expression of PTEN and inhibiting the phosphorylation of AKT. Similarly, MT-Exo significantly promoted the healing of diabetic wounds by inhibiting inflammation, thereby further facilitating angiogenesis and collagen synthesis in vivo.

**Conclusions:**

MT-Exo could promote diabetic wound healing by suppressing the inflammatory response, which was achieved by increasing the ratio of M2 polarization to M1 polarization through activating the PTEN/AKT signalling pathway, and the pretreatment of MT was proved to be a promising method for treating diabetic wound healing.

**Graphical abstract: MT-Exo promotes diabetic wound healing by regulating M1 and M2 macrophage polarization.:**

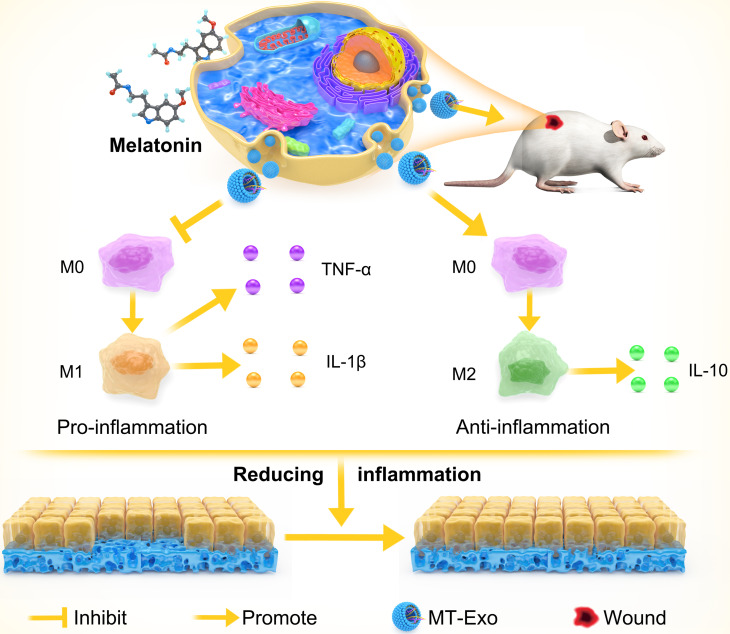

## Background

Delayed healing or non-healing surgical wounds caused by diabetes, which can lead to infection, affect the outcomes of surgery and may eventually become chronic wounds, afflicting many clinical surgeons worldwide. Current therapies for this issue include dressing changes, growth factor administration, cytokine administration and so on, but the effect is still not satisfactory [1, 2].

Recent researches showed that macrophage polarization plays an important role in the process of diabetic wound healing [3–5]. Classically activated macrophages (M1) and optionally activated macrophages (M2) are the two categories of macrophages [6]. M1 macrophages are featured by producing pro-inflammatory cytokines such as IL-1β, TNF-α and the subsequent inflammatory response may result in organ dysfunction [7, 8], while M2 macrophages are correlated with the production and secretion of anti-inflammatory cytokines, thereby alleviating the inflammatory response [9]. Notably, published studies suggested that increasing the M2 phenotype and decreasing the M1 phenotype were conductive to diabetic wounds repair [10, 11].

Mesenchymal stem cells (MSCs) have multi-differentiation potential and immunomodulatory capacities, which can significantly improve inflammation-related diseases [12]. Mesenchymal stem cells derived from human umbilical cord, for instance, were reported to instruct macrophage polarization to alleviate islet dysfunction in type 2 diabetic mice [13]. MSCs could act in a paracrine manner, including secreting growth factors, cytokines and exosomes, which have been extensively applied in the research of many diseases [14]. It has been reported that the paracrine function of MSCs could be applied to promote wound healing [15]. Exosomes, extracellular vesicles with diameters ranging from 30 to 150 nm, can transport different cargoes, such as proteins and nucleic acids, in a paracrine manner to exert different effects [16]. The potential benefits of exosomes methods over traditional cell-based therapies are that cell-free therapies based on exosomes can overcome side effects associated with the use of transplanted cells, such as immune rejection [17]. More importantly, exosomes secreted by MSCs could play a significant role in inhibiting M1 polarization and promoting M2 polarization to reduce inflammation. And the regulation of M2 polarization by MSC-derived exosomes could enhance skin wound healing [18]. MSCs can also promote polarization of M2 macrophages, thereby improving myocardial damage caused by diabetic cardiomyopathy [19].

Preconditioning, which could improve transplantation efficacy, is one of the key strategies to improve MSC function in vitro and in vivo for tissue engineering [20]. The biological functions of MSCs can be significantly enhanced by various pretreatment methods, such as cytokines, drugs, hypoxic conditions and physical factors. Also, pretreatment of MSCs can greatly enhance their potential for promoting IL-6-dependent M2b polarization which, in turn, promotes M2 polarization of macrophages [12]. Pretreatment of umbilical cord-derived MSCs with polyribonucleic acid can improve treatment efficiency in a polynitrotriphenylsulfonate-induced colitis mouse model [20, 21]. MSCs pretreated with vitamin E can increase the content of proteoglycans in the cartilage matrix, thereby achieving cartilage differentiation of MSCs [22].

Recently, many studies have demonstrated that pretreated MSCs acquired enhanced paracrine effects [23, 24]. For example, DMOG-pretreated hBMSC-derived exosomes can promote bone regeneration by targeting the AKT/mTOR pathway [25]. Salidroside-treated MSCs enhance the healing of diabetic wounds by promoting their paracrine function [26]. Fluoxetine pretreatment can also enhance the effects of MSCs on diabetic neuropathy [27].

Melatonin (MT) is a hormone that was first isolated from the pineal gland in 1959 and widely distributed in the body [28]. MT can promote the transfer of fat-derived exosomes to macrophages, further promoting M2 transformation and inhibiting fat inflammation [29]. Melatonin-pretreated exosomes can enhance the regeneration potential of MSCs derived from chronic kidney disease, improve rat kidney ischaemia-reperfusion injury and exert a therapeutic effect on acute liver ischaemia-reperfusion injury [30–32].

Therefore, in this research, we explored whether exosomes derived from hBMSCs pretreated with MT (MT-Exo) could inhibit M1 polarization, promote M2 polarization and further reduce inflammatory response and improve the regeneration of diabetic wounds.

## Materials and methods

### Cell culture

hBMSCs (P4) and RAW264.7 cell line were obtained from the Cell Bank of the Chinese Academy of Sciences and applied in our research, incubated in α-MEM and high glucose DMEM, respectively, consisting of 10% FBS (Gibco, Grand Island, NY, USA) and 1% penicillin and streptomycin. The experiment was divided into five groups: the PBS group, LPS group (lipopolysaccharide, 100 ng/mL, 24 h), LPS + Exo group (exosomes extracted from supernatant of hBMSCs without MT treatment), LPS + MT-Exo group (exosomes extracted from supernatant of hBMSCs treated with MT) and LPS + MT-Exo + SF1670 group (SF1670 is an inhibitor of PTEN). Subsequently, RAW264.7 cells were seeded into 24-well plates and cultured in the incubator (37 °C, 5% CO_2_).

### The characterization of hBMSCs

hBMSCs (P4) was obtained from the Cell Bank of the Chinese Academy of Sciences and applied in our research. For the characterization of hBMSCs, optical image for observing the adherence of hBMSC, flow cytometry and in vitro tri-lineage differentiation capacity were carried out. The surface markers CD105, CD90, CD73, CD45 and CD34 of hBMSC were verified by flow cytometry.

### Exosome extraction and purification

Exosomes, including Exo and MT-Exo derived from hBMSCs, were isolated from the supernatant via ultracentrifugation. Specifically, hBMSCs were pretreated with MT at a final concentration of 1 μmol/L in serum-free culture medium for 48 h. When the cell confluence reached 80%, the medium was harvested for centrifugation at 300*g* and 2000*g* to remove dead cells for 15 min and 20 min, respectively. Then, we filtered the harvested supernatant by a 0.22-μm filter (Micropore). Subsequently, Ultra-Clear™ tubes (Beckman Coulter, USA) were utilized for the filtration of supernatant at 100,000*g* for approximately 2 h twice. Finally, we used PBS for resuspending the required pellets before being kept at − 80 °C for further experiments.

### Exosome characterization

We observed the ultrastructure and shape of exosomes via transmission electron microscopy (TEM, JEM-1400). Likewise, the size distribution and nanoparticle concentration were evaluated by nanoparticle tracking analysis (NTA, ZetaView PMX 110, Particle Metrix). CD81, Tsg101, Alix and Calnexin were detected by Western blotting.

### ELISA for detecting inflammatory and anti-inflammatory factors

RAW264.7 cells incubated on 24-well plates were treated with PBS, LPS, LPS + Exo and LPS + MT-Exo for 24 h. Then, we collected the cell supernatants before measuring the levels of IL-1β, TNF-α and IL-10. The IL-1β ELISA kit, TNF-α ELISA kit and IL-10 ELISA kit were utilized for the detection of the levels of cytokines IL-1β, TNF-α and IL-10 in cell supernatants, respectively, according to the manufacturer’s specifications (Shanghai ExCell Biotechnology).

### Total RNA isolation and qRT-PCR analysis

For the quantification of the relative gene expressions of IL-1β, TNF-α and IL-10, Arg-1, and iNOS, qRT-PCR were applied. In short, TRIzol reagent (Invitrogen) was utilized for the extraction of total RNA from RAW264.7 cells. Later, complementary DNA (cDNA) was acquired by the reverse transcription of the extracted total RNA via the PrimeScript RT reagent Kit (Takara). Then, SYBR Green detection reagent (Takara) was applied for qRT-PCR analysis. Finally, we determined the relative expression levels by using the 2^-(△△CT)^ method and normalized them to 18S. Table S1 shows the primer sequences.

### Animal procedure

#### Air pouch assay in vivo

In this study, all the animal operations involved were permitted by the Animal Care and Ethics Committee of Shanghai Jiaotong University Affiliated Sixth People’s Hospital and were performed in accordance with established guidelines. The db/db mice were anaesthetized via intraperitoneal injection of 0.6% sodium pentobarbital and subcutaneously intraperitoneal injection sterilized air for establishing an air pouch model. At the same time, Exo and MT-Exo were also injected subcutaneously for observing the anti-inflammatory effect. Four days later, 2 mL of saline was used for washing the subcutaneous pouch for obtaining inflammatory cells. Subsequently, flow cytometry was utilized for determining the percentage of M1 and M2 macrophages. The M1 and M2 macrophages were labelled with AF647 (CCR7) and PERCP-CY5.5 (CD206) anti-mouse antibodies, respectively.

### Diabetic rat model establishment in vivo

Fifty-four Sprague-Dawley (SD) rats (250 g ± 10 g; 8 weeks old; male) were used for this operation. Diabetic models were generated by intraperitoneal injection of streptozotocin (STZ). Rats with fasting blood glucose levels over 11.1 mmol/l were selected for the operation. Subsequently, the rats were anaesthetized via intraperitoneal injection of 0.6% sodium pentobarbital (10 mL/kg). After anaesthesia, one circular full-thickness dermal defect with a diameter of 2 cm was aseptically created in the middle of the rat’s back and treated with PBS (Control), Exo and MT-Exo by multisite subcutaneous injection (at least six sites per wound). After the operation, a skin patch was applied to cover the circular wound defects. All rats that underwent surgery gained access to abundant food and water and were treated with penicillin injected intramuscularly for approximately 3 days. In the end, the rats were transferred to the biosafety facility after anaesthesia was discontinued. At days 0, 3, 7 and 14 after surgery, the wounds were imaged via a digital camera. Image J (NIH) was applied to determine the healing level of the wound dimension. The wound closure rate (WCR, %) was calculated as follows: WCR = [(*S*_0_ − *S*_*t*_)/*S*_0_] × 100%. *S*_0_ is the initial wound dimension and *S*_*t*_ is the wound dimension at each time point.

### Histological analysis

After the sacrifice was carried out by intraperitoneal injection of an overdose of 0.6% sodium pentobarbital, wound sections were obtained and fixed in 4% paraformaldehyde at day 7 and 14, postoperatively. The harvested tissues were gradually dehydrated and embedded in paraffin. Then, the paraffin-embedded tissues were sliced into 5-μm-thick sections, which were used for haematoxylin and eosin (H&E) and Masson’s trichrome staining for the observation of the neoepithelium length and the degree of collagen maturity, respectively.

### Immunohistochemistry staining analysis

For immunohistochemistry staining (IHC), the harvested sections were deparaffinized and rehydrated. Subsequently, the deparaffinized sections were processed with secondary antibody and ABC complex following incubation with the α-SMA primary antibody (1:250, Abcam). Finally, the samples were visualized by the chromogenic substrate diaminobenzidine (DAB) substrate. An optical microscope (Olympus IX 70, Tokyo, Japan) was applied to obtain images of the stained sections.

### Immunofluorescence analysis

After the harvested sections were deparaffinized and rehydrated, 1.5% goat serum (Merck-Millipore) was utilized for blocking for immunofluorescence (IF). Alexa Fluor 488 and Cy3-conjugated secondary antibody and DPAI (Sigma-Aldrich) were applied for incubation for visualization following treatment with the primary antibody CD31 (1:200, Abcam), as well as alpha-smooth actin (α-SMA) (1:50, Abcam), the angiogenesis markers. Then, a fluorescence microscope was used for the fluorescence observation. For the quantitative image analysis of the newly developed blood vessels, Image J (NIH Image) was performed.

### Microfil perfusion

Microfil perfusion was used for the evaluation of neovascularization. The chest of the rats was open to expose the heart and relevant arteries after being anaesthetized by 0.6% phenobarbital 14 days after the surgery. An indwelling needle was employed for penetration of the left ventricle. Heparinized saline and Microfil (Microfil MV-122; Flow Tech, Carver, MA) perfusate were injected through the indwelling needle. All the samples were immediately placed at 4 °C to induce the polymerization of the Microfil agent. After the polymerization, the samples were scanned by micro-CT (Skyscan 1176, Belgium) and the number of neovasculars was calculated using Image J (NIH Image).

### Western blotting

To evaluate protein expression, Western blotting was performed. In short, RAW264.7 cells were harvested and lysed in prechilled RIPA buffer containing a phosphatase inhibitor cocktail and PMSF for 10 min on ice. The lysates were diluted with 5× loading buffer. After that step, the dilution was boiled at 95 °C for approximately 10 min. Gradient SDS-PAGE (10–20%) was applied for separation of proteins. Then, the extracted proteins were transferred from SDS-PAGE onto a PVDF membrane (Merck-Millipore) before being blocked with 5% (w/v) nonfat milk. Finally, the PVDF membrane was incubated with primary antibodies overnight and secondary antibodies for 1 h. An ECL substrate kit (Thermo Fisher Scientific Inc., USA) was utilized for the visualization of protein bands in the membrane.

### Statistics

One-way ANOVA and Student-Newman-Keuls post hoc tests were used for the assessment of the statistical significance. GraphPad software was utilized for the statistical analysis of the mean ± SEM. *P* values < 0.05 were deemed significant.

## Results

### Characterization of hBMSCs

The identification of hBMSCs was confirmed adherence ability by optical images, surface markers by flow-cytometric analysis and tri-lineage differentiation (Figure S1). It can be observed that hBMSCs could adhere to plastic culture disk and show a spindle-like morphology (Figure S1a). Trilineage differentiation including osteogenesis, adipogenesis and chondrogenesis was performed to assess their pluripotency (Figure S1b-d). The osteogenesis was evaluated by Alizarin Red staining after 2 weeks of differentiation. Adipogenesis was assessed by detecting the formation of small cytoplasmic lipid droplets by Oil Red O staining after 3 weeks of differentiation. Chondrogenesis differentiation potential was determined by polysaccharides and proteoglycans through Alcian Blue staining after 3 weeks of differentiation. Also, flow cytometry showed > 95% positive for the surface markers CD105, CD90, CD73 and < 2% negative for the surface markers CD45 and CD34 (Figure S1e). All the above-mentioned results confirmed that the hBMSCs possessed MSC properties and pluripotency.

### The isolation and characterization of exosomes

**Fig. 1:**
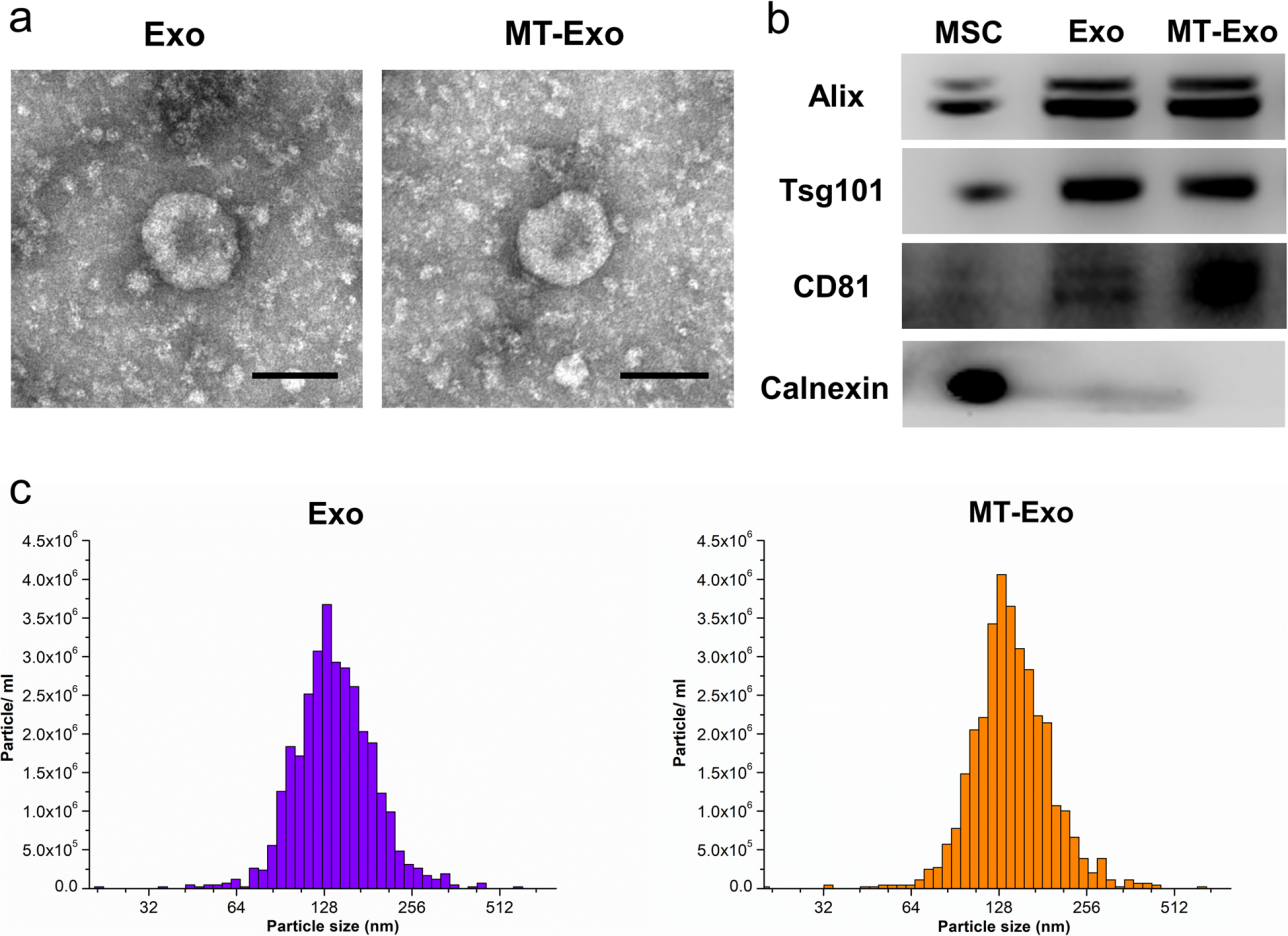
The characterization of Exo and MT-Exo. **a** The morphology of Exo and MT-Exo was analysed by TEM. Scale bar = 100 nm. **b** The surface markers (Alix, Tsg101, CD81 and Calnexin) of Exo and MT-Exo were evaluated by Western blotting. hBMSCs were used as a control and Calnexin was used as a negative control. **c** The diameter and concentration of exosomes were measured by NTA analysis (*n* = 3)

Exosomes were isolated from the supernatant of hBMSCs treated with or without MT via ultracentrifugation. TEM, Western blotting and NTA were carried out to verify the exosomes. TEM was used to visualize the morphology of Exo and MT-Exo. We observed that both types of exosomes were oval bilayer lipid membrane vesicles with a diameter of approximately 120 nm with no significant difference between them (Fig. 1a). CD81, Tsg101, Alix and Calnexin were detected by Western blotting for Exo and MT-Exo, showing no significant difference between them (Fig. 1b). NTA analysis illustrated that the size of the Exo and MT-Exo ranged from 30 to 150 nm with mean diameters of 130 nm and 125 nm, respectively (Fig. 1c). At the same time, the concentrations of Exo and MT-Exo were approximately 7.0 × 10^8^ and 7.5 × 10^8^, respectively, showing no significant difference.

### MT-Exo inhibited the inflammatory response by increasing the ratio of M2 polarization to M1 polarization in vitro

**Fig. 2:**
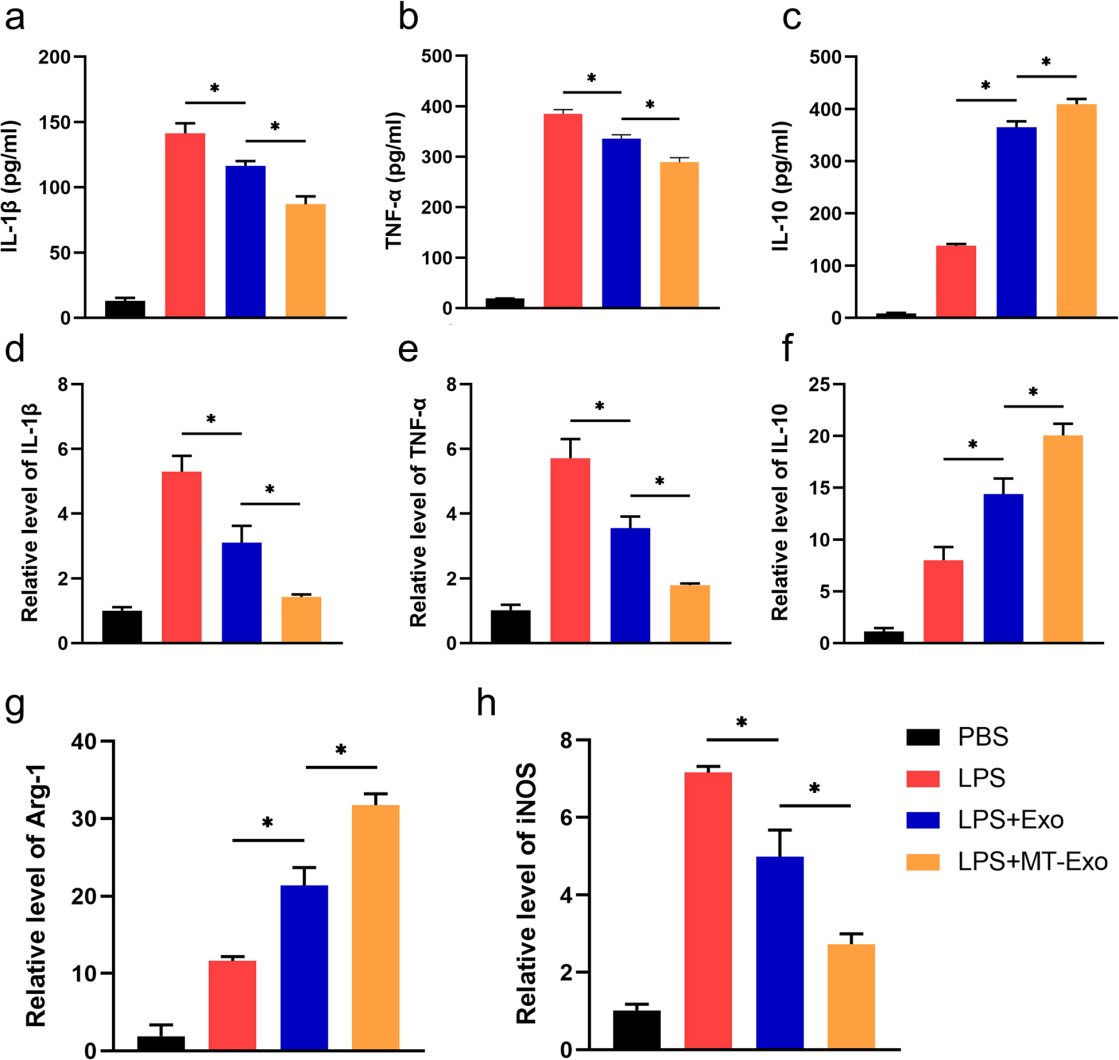
MT-Exo inhibited the inflammatory response by increasing the ratio of M2 polarization macrophages to M1 polarization in vitro. RAW264.7 cells were treated with PBS, LPS (100 ng/mL), LPS + Exo and LPS + MT-Exo for 24 h. ELISA was performed to detect the concentrations of **a** IL-1β, **b** TNF-α and **c** IL-10 from the supernatants. The relative gene expressions of **d** IL-1β, **e** TNF-α, **f** IL-10, **g** Arg-1 and **h** iNOS were detected (*n* = 3, *p < 0.05)

For the evaluation of the polarization state of macrophages after treatment with equivalent PBS, LPS, Exo and MT-Exo, we detected the anti-inflammatory secreted cytokines IL-10 by ELISA and the relative gene expression levels of Arg-1 and IL-10 by qRT-PCR. Also, we detected the anti-inflammatory secreted IL-1β, TNF-α and the relative gene expression levels of IL-1β, TNF-α and iNOS after incubation 24 h later. By ELISA, we observed that the secretion of IL-1β and TNF-α in the Exo group was significantly reduced in comparison with that in the PBS and LPS groups, and these pro-inflammatory cytokines in the MT-Exo group were also significantly decreased in comparison with those in the Exo group (Fig. 2a, b). In contrast, we noticed that IL-10 secretion in the Exo and MT-Exo groups was significantly increased than that in the PBS and LPS groups, and these anti-inflammatory cytokines in the MT-Exo group were also significantly enhanced than those in the Exo group (Fig. 2c). For evaluating the relative gene expression of pro-inflammation and anti-inflammation genes, qRT-PCR was performed. The results indicated that the relative levels of iNOS, IL-1β and TNF-α in the Exo and MT-Exo groups were significantly decreased in comparison with those in the PBS and LPS groups, and the relative level in the MT-Exo group was also significantly increased than that in Exo group (Fig. 2d, e and h). We found that the relative levels of Arg-1 and IL-10 in the Exo and MT-Exo groups were significantly increased in comparison with those in the PBS and LPS groups, and the relative level in the MT-Exo group was also significantly increased in comparison with that in the Exo group (Fig. 2f and g). All the above-mentioned data illustrated that Exo could increase the ratio of M2 polarization to M1 polarization and MT could augment this effect, showing its potential in inflammation-related diseases.

### MT-Exo inhibited the inflammatory response by increasing the ratio of M2 polarization to M1 polarization in vivo

**Fig. 3:**
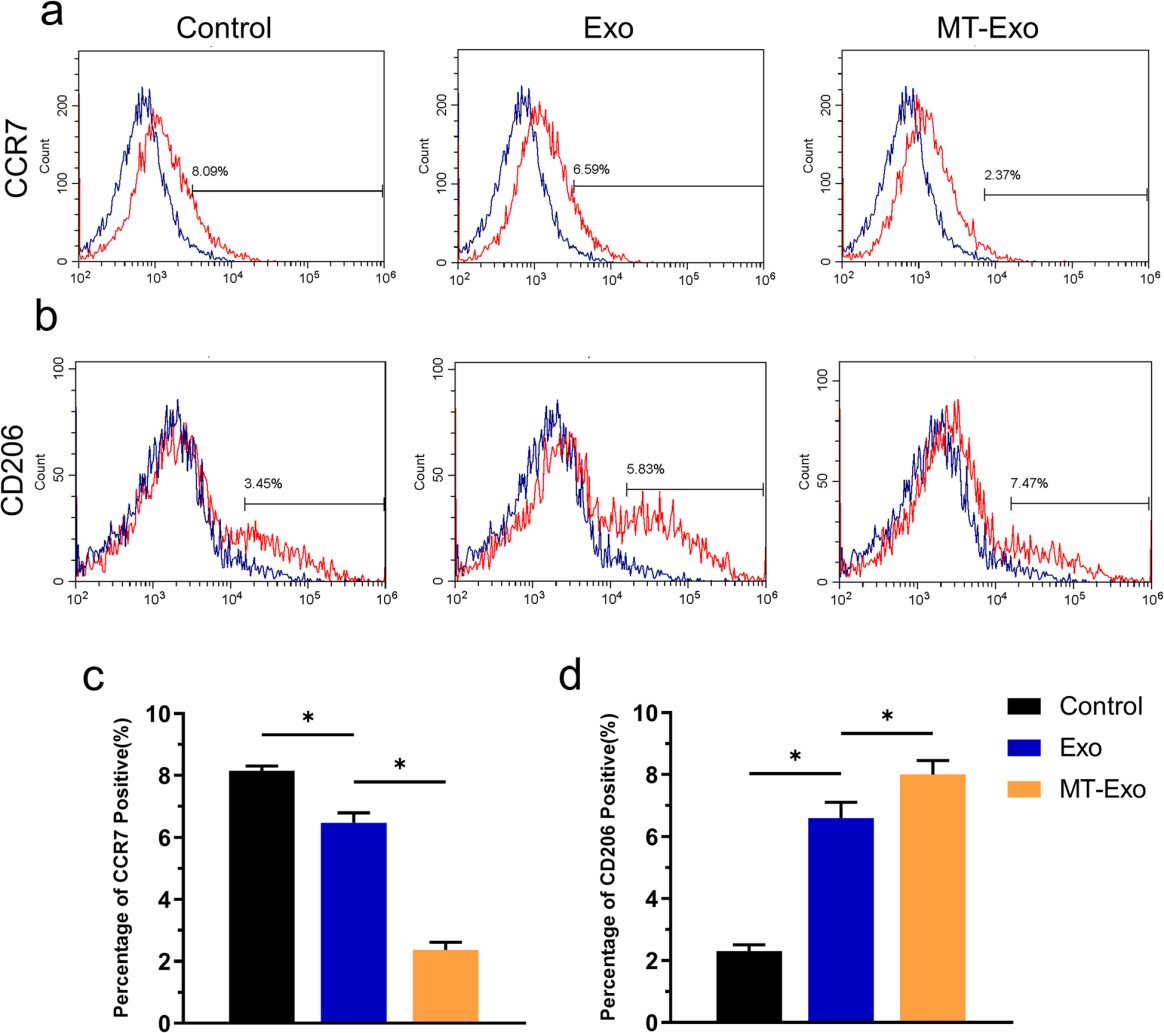
MT-Exo increased the ratio of M2 to M1 polarization in vivo. **a**, **b** Representative images of macrophage polarization of surface markers (CCR7 and CD206) of RAW264.7 by flow cytometry analysis (*n* = 3, *p < 0.05). **c** The quantitative analysis of percentage of CCR7 positive cells of the Control, Exo and MT-Exo group (*n* = 3, *p < 0.05). **d** The quantitative analysis of percentage of CD206 positive cells of the Control, Exo and MT-Exo group (*n* = 3, *p < 0.05)

For the evaluation of the polarization state affected by MT-Exo of macrophages in vivo, we used diabetic db/db mice to establish air pouch models and detect the macrophages by flow cytometry. On one hand, it can be observed that the CCR7 positive cells, diaplaying the percentage of M1 polarization macrophages, were high in the Control group at the beginning. But after the application of Exo and MT-Exo, the percentage of CCR7 positive cells was significantly decreased and MT-Exo had stronger effect compared with Exo (Fig. 3a, c). On the other hand, CD206 positive cells, suggesting the percentage of M2 polarization macrophages, were low in the Control group at the beginning. But after the application of Exo and MT-Exo, the percentage of CD206 positive cells was significantly increased and MT-Exo had stronger effect compared with Exo (Fig. 3b and d).

### Verification of the diabetic rat model

We verified the successful establishment of a diabetic model in STZ-treated SD rats. Diabetes is characterized by increased blood glucose, increased food and water intake and decreased body weight. The fasting blood glucose (FBG) of the STZ group was stable soon after the injection of STZ but significantly increased on the 5th day in comparison with that of the Control group (Figure S2a). On the 10th day, the mean FBG was over 11.1 mmol/L, which met the blood glucose level requirements of diabetes. The body weight of the STZ group was significantly decreased on the 5th and 10th day in comparison with that of the Control group (Figure S2b). For assessing the food and water intake, we can conclude that the rats in the STZ group consumed considerably more food and water than those in the Control group (Figure S2c-d). All the data suggested the successful establishment of the diabetic model.

### MT-Exo improves diabetic wound healing in SD rats in vivo

**Fig. 4:**
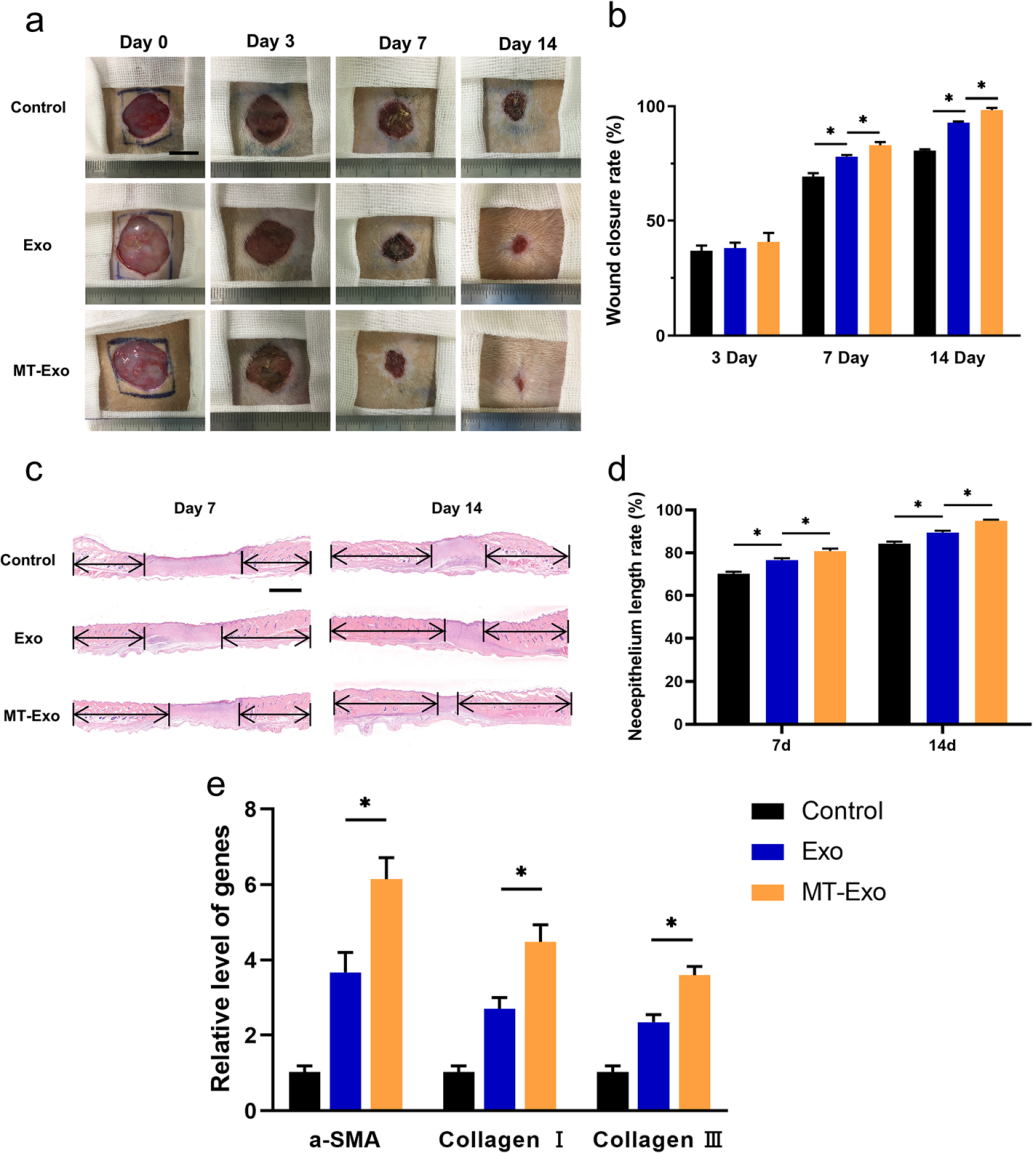
MT-Exo expedited diabetic wound healing in vivo. **a**, **b** Optical images and related quantification of the wound closure rate of full-thickness dermal defects in the Control group, Exo group and MT-Exo group at day 0, 7 and 14 after the skin operation (*n* = 3, *p < 0.05, Scale bar = 10 mm). **c**, **d** H&E staining images and related quantification of total neoepithelium length in the Control group, Exo group and MT-Exo group at days 7 and 14 (*n* = 3, *p < 0.05). **e** The relative gene expression of the angiogenesis-related gene α-SMA and collagen synthesis-related genes Collagen I and III in the Control, Exo, and MT-Exo groups (*n* = 3, *p < 0.05)

To evaluate the effect of MT-Exo on diabetic wound healing, we performed full-thickness dorsal wound surgery in STZ-induced diabetic SD rats. We observed optical images of diabetic wounds treated with PBS, Exo and MT-Exo for 0, 3, 7 and 14 days (Fig. 4a and b). The sizes in the Exo and MT-Exo groups were significantly reduced in comparison with that in the Control group at day 7, and the reduction of wound area in the MT-Exo group was significantly greater in comparison with that in the Exo group. This trend may be attributed to the anti-inflammatory effect of MT-Exo on macrophages by promoting M2 and inhibiting M1 polarization at the beginning of the wound healing process, which shortens the transition time from the inflammation stage to the tissue formation stage. Similarly, there appeared to be a similar trend at day 14. For the evaluation of neoepithelium length, H&E staining was performed (Fig. 4c and d). Neoepithelium length was labelled by the black arrow in Fig. 4c and the neoepithelium length rate was calculated by neoepithelium length/total wound length. The neoepithelium length in the Exo and MT-Exo groups was significantly increased in comparison with that of the Control group, and the increase of neoepithelium length in the MT-Exo group was significantly greater in comparison with that in the Exo group. For assessing the neovascularization and collagen synthesis, qRT-PCR was carried out by the skin tissues of wound after the rats were sacrificed (Fig. 4e). We found that the angiogenesis-related gene α-SMA was upregulated and the collagen-related genes Collagen I and III were significantly upregulated in both the Exo and MT-Exo groups in comparison with the Control group, and the relative expression level in the MT-Exo group was significantly increased in comparison with that in the Exo group.

### MT-Exo improves angiogenesis and collagen synthesis in diabetic rats in vivo

**Fig. 5:**
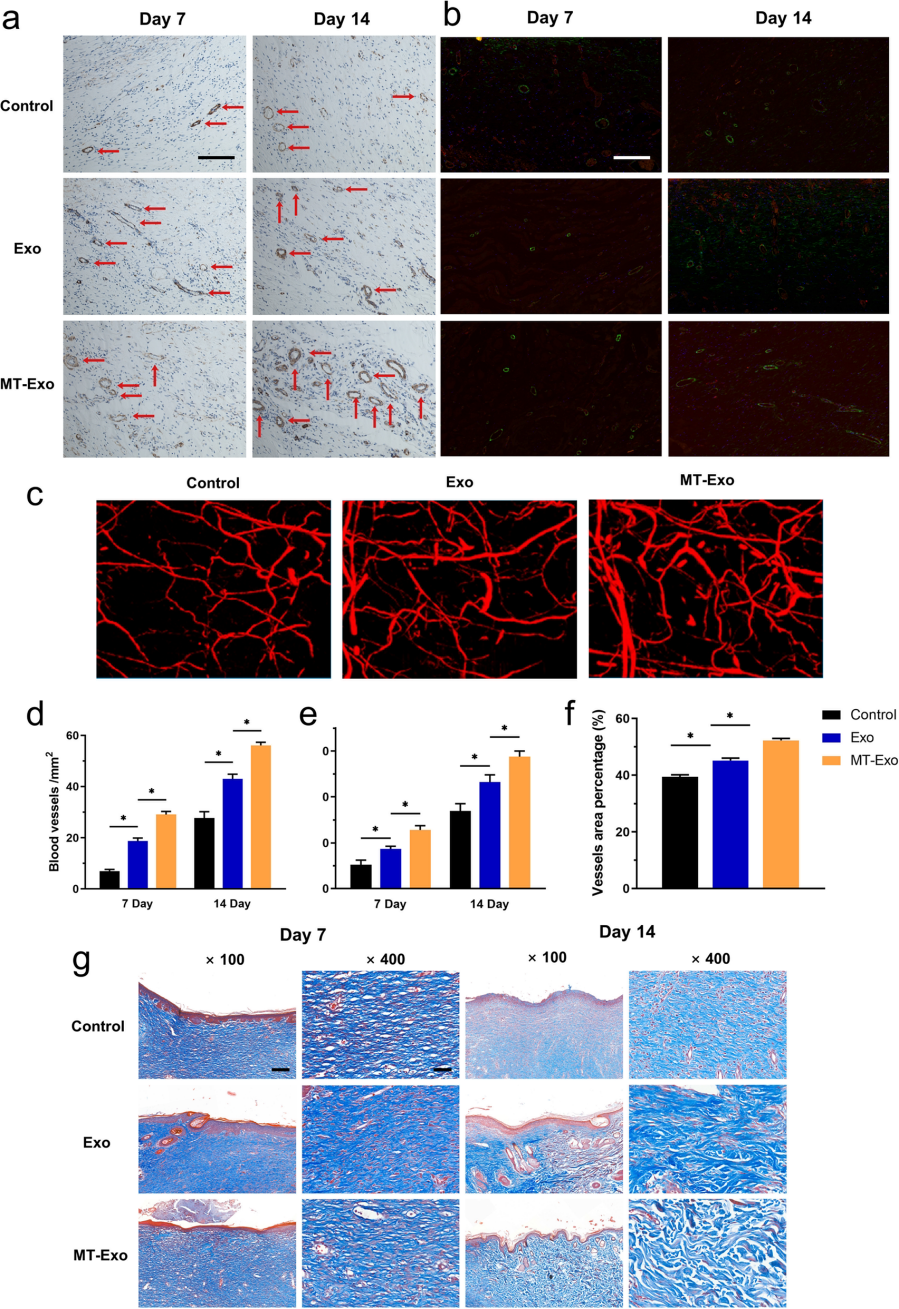
MT-Exo improves angiogenesis and collagen synthesis in diabetic rats in vivo. **a** The assessment of the neovessels in the Control group, Exo group and MT-Exo group by α-SMA IHC at day 7 and 14 (red arrows display neovessels, Scale bar = 100 μm). **b** IF evaluation for CD31/α-SMA in the Control group, Exo group and MT-Exo group at days 7 and 14 (Scale bar = 100 μm). **c** The evaluation of neovasculars via the Microfil imaging method. **d** Quantification of the number of neovessels per field at day 7 and 14 by IHC (*n* = 3, *p < 0.05). **e** Quantification of the number of neovessels per field at day 7 and 14 by IF. **f** Quantification of the number of neovasculars per field at day 7 and 14 by Microfil. **g** Masson’s trichrome staining at day 7 and 14 post-operationally (Scale bar = 200 μm for 100× and 50 μm for 400×)

Subsequently, we assessed angiogenesis and collagen synthesis in vivo. We found from IHC (α-SMA), (IF) CD31/α-SMA and Microfil perfusion that the amount of newly developed blood vessels in the Exo and MT-Exo groups were significantly greater in comparison with that in the Control group at day 7 and 14, and the MT-Exo group had more vessels than the Exo group (Fig. 5a-f). Similarly, we observed from Masson trichrome staining that the collagen fibres in the MT-Exo group were significantly thicker in comparison with those in the Exo and Control groups at day 7 and 14, and the MT-Exo group had much thicker collagen fibres than the Exo group (Fig. 5g). As a consequence, we can further conclude that Exo could promote angiogenesis and collagen synthesis under high-glucose conditions in vivo, which is characterized by delayed inflammation, and MT could augment this effect.

### MT-Exo increased the ratio of M2 polarization to M1 polarization by activating the PTEN/AKT signalling pathway

**Fig. 6:**
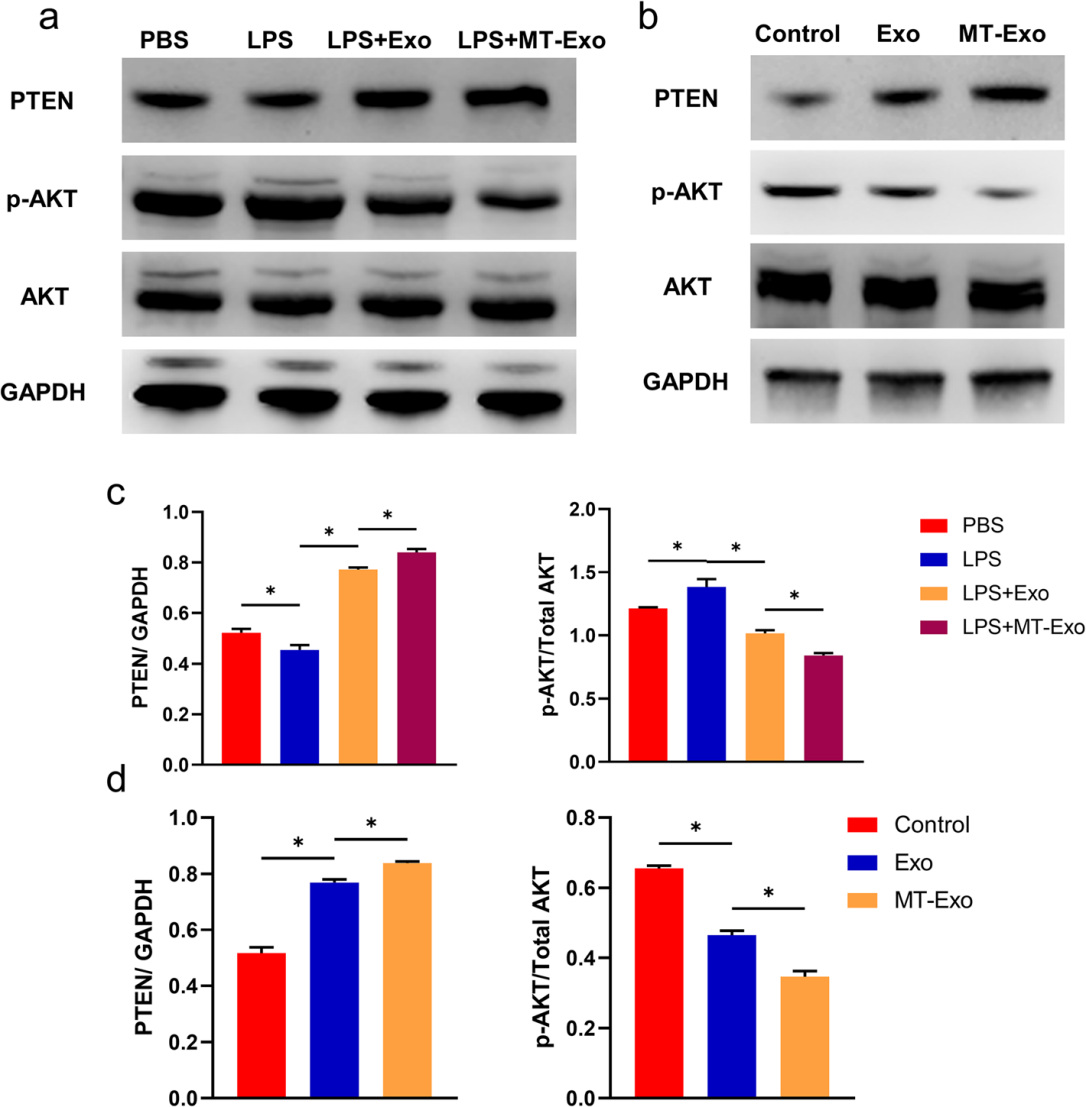
MT-Exo suppressed inflammation by activating the PTEN/AKT signalling pathway. **a** The expressions of PTEN, phosphorylation of AKT, AKT (total AKT) and GAPDH were tested in RAW264.7 cells after treatment with PBS, LPS (100 ng/mL), LPS + Exo and LPS + MT-Exo for 24 h by Western blotting. GAPDH was utilized as an internal reference. **b** The expressions of PTEN, phosphorylation of AKT, AKT (total AKT) and GAPDH were tested in vivo after treatment with PBS, Exo and MT-Exo by Western blotting. GAPDH was utilized as an internal reference. **c** The quantification of the greyscale values of PTEN/ GAPDH and p-AKT/AKT in vitro (*n* = 3, *p < 0.05). **d** The quantification of PTEN/ GAPDH and p-AKT/ AKT by Western blotting in vivo (*n* = 3, *p < 0.05)

To assess the underlying mechanisms of MT-Exo’s anti-inflammatory effect, we determined PTEN and the phosphorylation levels of AKT in vitro and in vivo, which plays a vital role in macrophage polarization. In vitro, the phosphorylation of AKT in the LPS group showed the most significant growth in comparison with that in the PBS group, while the phosphorylation of AKT in the Exo and MT-Exo groups was significantly reduced in comparison with that of the LPS group, which verified the enhancement of M2 polarization and suppression of M1 polarization. Meanwhile, the expression of PTEN in the Exo and MT-Exo groups, which negatively regulated the phosphorylation levels of AKT, was significantly increased in comparison with that of Control group, which verified the suppression of M1 polarization and augmentation of M2 polarization (Fig. 6a and c). In vivo, the phosphorylation of AKT in the Exo and MT-Exo groups was also significantly decreased compared with that in the Control group. Similarly, the expression of PTEN was significantly increased compared with that of Control group (Fig. 6b and d).

**Fig. 7:**
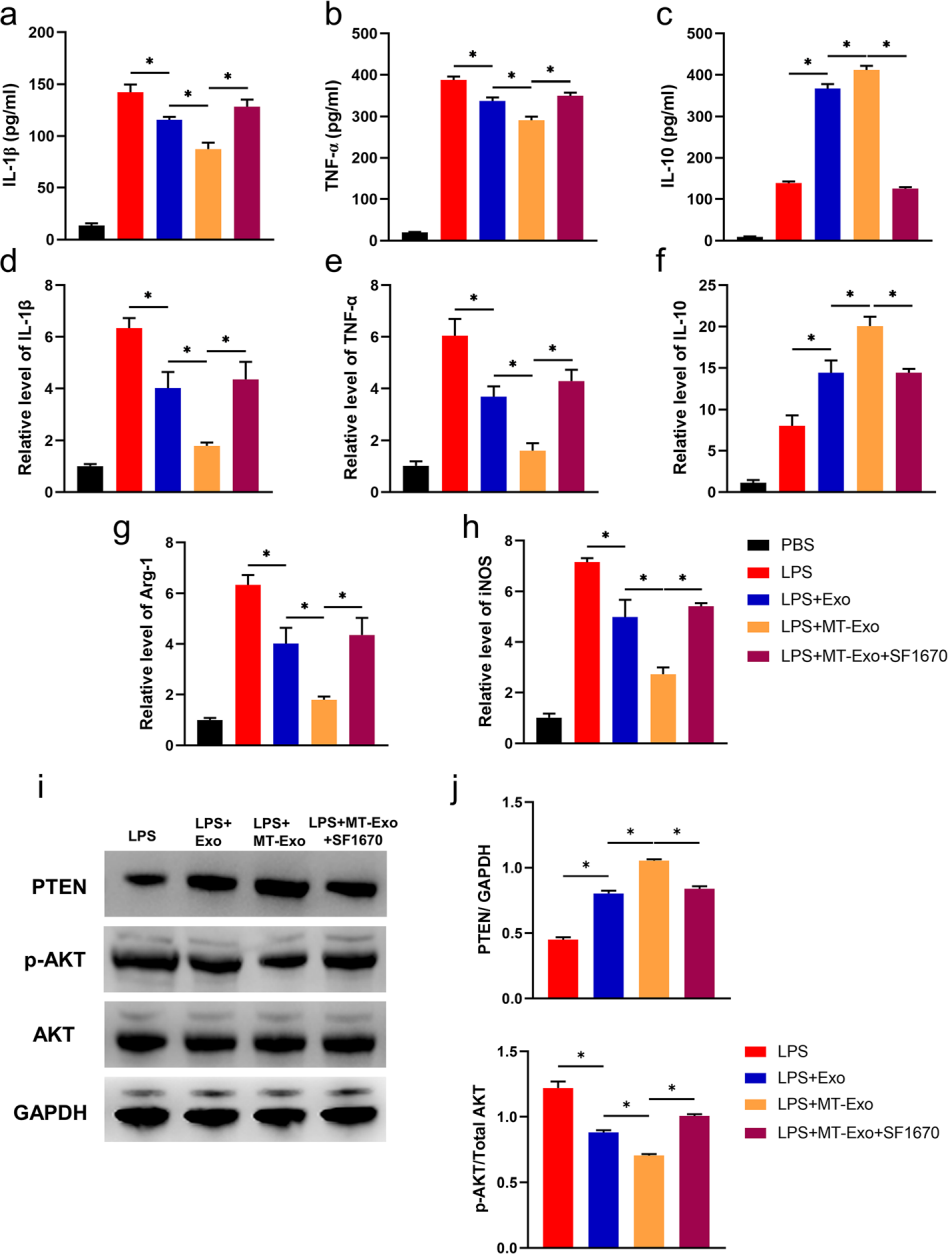
PTEN inhibitor SF1670 antagonized the anti-inflammatory effect of MT-Exo. RAW264.7 cells were treated with PBS, LPS (100 ng/mL), LPS + Exo, LPS + MT-Exo and LPS + MT-Exo + SF1670 for 24 h. Then, ELISA was performed to detect the concentrations of **a** IL-1β, **b** TNF-α and **c** IL-10 from the supernatants. The relative gene expressions of **d** IL-1β, **e** TNF-α, **f** IL-10, **g** Arg-1 and **h** iNOS were detected via qRT-PCR (*n* = 3, *p < 0.05). **i** The expression of PTEN, phosphorylation of AKT, AKT (total AKT) and GAPDH were also tested. GAPDH was utilized as an internal reference. **j** The quantification of PTEN/GAPDH and p-AKT/AKT by Western blotting (*n* = 3, *p < 0.05)

To further validate the effect of the PTEN/AKT signalling pathway on the regulation of macrophage polarization, we applied the PTEN inhibitor SF1670. After inhibition, we observed that the MT-Exo-SF1670 group showed significantly higher secretion of IL-1β and TNF-α and significantly reduced IL-10 in comparison with the MT-Exo group (Fig. 7a-c). Furthermore, the MT-Exo-SF1670 group demonstrated significantly higher relative gene expression levels of IL-1β, TNF-α and iNOS and significantly reduced gene expression levels of Arg-1 and IL-10 compared with the MT-Exo group (Fig. 7d-h). The expression of PTEN was enhanced significantly in the Exo and MT-Exo groups compared with the LPS group and inhibited significantly in the MT-Exo-SF1670 group. The phosphorylation of AKT was also significantly enhanced in the MT-Exo-SF1670 group in comparison with the MT-Exo group (Fig. 7i and j). The above data illustrated that SF1670 weakened the anti-inflammatory effect mediated by M2 polarization and augmented the pro-inflammatory effect via M1 polarization by inhibiting the function of PTEN and enhancing the phosphorylation of AKT.

## Discussion

In this study, we determined whether MT-Exo could play a crucial anti-inflammatory role in diabetic wound healing and its underlying mechanisms. Our research demonstrated that MT-Exo inhibited the activation of the PI3K/AKT pathway by promoting the expression of PTEN to regulate M1 and M2 macrophage polarization, thereby inhibiting the inflammatory phase of diabetic wound healing in STZ-induced SD diabetic rats, which facilitated a quicker transition from the inflammation phase to the tissue regeneration phase. Our results suggested that MT-Exo was an exceptionally meaningful and promising approach for the healing of diabetic wounds.

Wound healing could be divided into four steps: haemostasis, inflammation, hyperplasia and remodelling [33, 34]. All processes are intertwined, and a prolonged inflammation period will cause adverse effects on the subsequent regeneration. Excessive inflammation and vascular lesions due to hyperglycaemia around diabetic wounds prolong the inflammatory period and delay the wound healing process. Mirza et al. showed that the IL-1β was enhanced in diabetic wounds [35]. Delayed wound healing can strongly increase the risk of wound infection, which requires debridement in clinical practice, further disrupting the vascular bed around the wound and, in turn, worsening the healing process, resulting in a vicious cycle [36, 37]. Anti-inflammatory and shortening the inflammatory period as soon as possible are the keys to breaking this vicious circle. Previous studies have shown that the inhibition of the IL-1β pathway leads to accelerated wound healing in mice by inducing the transition of macrophages from an inflammatory phenotype to a repair phenotype [38]. According to our results, MT pretreatment could endow MSC-derived exosomes with better biological effects, increasing the ratio of M2 polarization to M1 polarization, thereby inhibiting inflammation and promoting tissue repair.

MSCs are characterized by self-renewal, undifferentiation and the ability to differentiate into multiple cell lineages [39, 40]. Therapies based on MSCs have been proved to have good efficacy in many diseases such as imperfecta, fractures, brain trauma, stroke and myocardial infarction in both animal models and clinical trials due to their convenient isolation, low immunogenicity and anti-inflammatory properties [41, 42]. In addition, it has been demonstrated that MSCs facilitate skin repair by regulating the inflammatory response, thereby promoting the formation of favourable blood vessels and collagen synthesis [43]. Moreover, many researchers believed that MSCs attribute their therapeutic effect mainly to paracrine signalling, namely, secreting bioactive molecules that influence the biological functions of neighbouring cells [42, 44]. However, there are many potential risks of MSC transplantation therapies, such as immune rejection and ectopic tissue formation [25]. It has also been reported that intra-arterial administration of MSCs led to the occurrence of myocardial micro-infarction and pulmonary embolism [45, 46]. Thus, we applied MSC-derived exosomes instead of MSCs, avoiding the above-mentioned risks. In this study, we established a reliable diabetic wound in the back of rats, and our data indicated that MT-Exo suppressed the inflammatory response, availed angiogenesis and collagen synthesis, and ultimately promoted diabetic wound healing.

It is imperative to inhibit M1 polarization because increased M1 macrophages have been reported to aggravate the progression of chronic ulcers [47, 48]. Some inflammation-associated factors were increased during the process of diabetic wound healing, thereby prolonging the inflammation phase, and contributing to delayed wound healing [49, 50]. For instance, IL-1β and TNF-α were increased in chronic wounds, leading to elevated metalloproteinases, which excessively degraded the local extracellular matrix and harmed the cell migration [51].

M2 polarization of macrophages in vascular conditions surrounding diabetic wounds is beneficial for angiogenesis and collagen synthesis [52]. Insufficient local angiogenesis is deemed an important cause of poor healing of chronic wounds [53]. Compared with acute wounds, higher expression of anti-angiogenic proteins (such as myeloperoxidase) have been found in chronic wounds in diabetic patients, while pro-angiogenic stimuli (such as extracellular superoxide dismutase) are usually reduced [54]. Specifically, M2 macrophages promote angiogenesis by releasing pro-angiogenic mediators [55]. In the proliferation phase of wound healing, M2 macrophages produce a plethora of pro-angiogenesis factors such as VEGF, FGF and EGF [56]. Additionally, IL-19 improves angiogenesis of ischaemic state via direct regulation of macrophage polarization [57]. p38αMAPK/MAPKAP Kinase 2 (MK2) could promote M2 macrophage polarization, thus promoting tumour progression [58]. Our research showed that MT-Exo enhanced macrophage M2 polarization and further facilitated blood vessel regeneration in vivo, which is a highly favourable factor for damaged diabetic wounds.

Several studies have shown that pretreatment with drugs increased the expression of related anti-inflammatory proteins or factors not only in stem cells but also in their exosomes. Huang et al. reported that atorvastatin pretreatment can upregulate lncRNA H19 in MSCs and their secreted exosomes, which could be utilized for treating acute myocardial infarction in rats by inhibiting the inflammatory factors, along with increased release of VEGF and enhanced MSC-mediated cardioprotective effect [59]. Ding et al. reported that BMSCs pretreated with deferoxamine upregulated the expression of miR-126 both in cells and secreted exosomes, reinforcing the angiogenic ability of endothelial cells, which further facilitating the healing of diabetic wounds [60].

For investigating the potential mechanism of the inhibitory effect of MT-Exo on inflammation, we evaluated the expression of key proteins in macrophage polarization-related pathways. AKT is a key protein that promotes M1 polarization, and the phosphorylation of AKT can promote macrophage M1 polarization and inhibit M2 polarization, thereby promoting the inflammatory response. PTEN is able to antagonize the activity of PI3K by converting PI (3,4,5) P3, which plays a leading role in the phosphorylation of AKT, into PI (4,5) P2, achieving negative regulation of the PI3K/Akt signalling pathway [61, 62]. Western blotting demonstrated that in comparison with the Control group, MSC-Exo could significantly increase the expression of PTEN, and MT can enhance this effect, thereby suppressing the phosphorylation of AKT and promoting macrophages to M2 polarization. The phosphorylation of AKT and the inflammatory response are reinforced after the application of the PTEN inhibitor SF1670. The inhibitory effect of MT-Exo on AKT phosphorylation was significantly weakened, and AKT phosphorylation was enhanced, which showed similar trends in comparison with that of the LPS group, consistent with the conclusions of previous findings [63].

Exosome incubated with different treatments can exert different potential paracrine effect on cell-cell communication, which may be responsible for the effect of of MT-treated exosome on macrophages. It has been reported that MT could increase the ratio of M2 to M1 by elevating adipose-derived exosomal α-ketoglutarate (αKG) level in macrophages, thus alleviating adipose inflammation [64]. Resistin delivered from adipocyte-derived exosome could trigger endoplasmic reticulum stress, which contributes to the consequent hepatic steatosis via the crosstalk to the liver, while MT could significantly decrease the adipocyte-derived exosomal resistin and remarkably ameliorated hepatic steatosis [29]. In addition, exosomes derived from MT-treated vascular smooth muscle cells could attenuate vascular calcification and ageing by regulating exosomal components miR-204/miR-211 [65]. Liu et al. also reported that lithium-containing biomaterials could promote the upregulation of miR-130a in hBMSC-derived exosomes, thus enhancing the angiogenesis of endothelial cells [66]. Guo et al. reported that hypoxia could induce glioma to secrete exosomes with increased expression of miR-10a and miR-21 [67].

Therefore, we concluded that MT-Exo could promote M2 polarization and inhibit M1 polarization by upregulating PTEN expression, thereby inhibiting the phosphorylation of AKT, which suppresses inflammatory responses. Ultimately, the prolonged inflammatory period in chronic wounds was shortened, thereby improving the diabetic wound repair.

## Conclusion

Our research demonstrated that MT-Exo could suppress inflammation through increasing the ratio of M2 polarization to M1 polarization by activating the PTEN/AKT signalling pathway and could enhance diabetic wound healing. Meanwhile, MT-Exo could promote angiogenesis and collagen synthesis in vivo due to the improvement of excessive inflammation status. We believe MT-Exo is a satisfactory candidate for diabetic wound healing and may be applicable in clinical practice. However, the detailed mechanism regarding the effect of MT-Exo needs to be elucidated, which is a limitation of this study. We expect to explore this mechanism further in future studies.

## Supplementary information

**Additional file 1: Figure S1.** The identification of hBMSCs. a: hBMSCs adhered to plastic culture disk. Scale bar = 500 μm. b-d: The osteogenesis, adipogenesis, and chondrogenesis differentiation of hBMSC respectively. Scale bar = 200 μm, 100 μm, and 200 μm repectively. e: The surface markers CD105, CD90, CD73, CD45, CD34 of hBMSC by flow cytometry. **Figure S2.** The verification of STZ-treated diabetic rat model. a The FBG of Control group and STZ group was detected via blood glucose test strips in SD rats on the 0, 5th,10th day. The rats without STZ injection was utilized as Control (*n* = 3, *p < 0.05). b The body weight of Control group and STZ group was measured by electronic weighing scale (*n* = 3, *p < 0.05). c The water intake and d food intake of Control group and STZ group was measured through electronic weighing scale (*n* = 3, *p < 0.05). **Table S1.** The RNA primers applied for qRT-PCR.

## Data Availability

The datasets used and/or analysed during the current study are available from the corresponding author on reasonable request.

## Abbreviations

hBMSC: Human bone marrow mesenchyme stem cell
MT: Melatonin
MSC: Mesenchyme stem cell
Exo: Exosome
MT-Exo: Melatonin-pretreated exosome
STZ: Streptozocin
TEM: Transmission electron microscope
NTA: Nanoparticle tracking analysis
