# Supplementary material for: Melatonin-stimulated MSC-derived exosomes improve diabetic wound healing through regulating macrophage M1 and M2 polarization by targeting the PTEN/AKT pathway
Source: Stem Cell Res Ther. 2020 Jun 29;11:259. doi: 10.1186/s13287-020-01756-x (PMC7322868; doi:10.1186/s13287-020-01756-x)
Supplement: Supplementary file 1 — Additional file 1: Figure S1. The identification of hBMSCs. a: hBMSCs adhered to plastic culture disk. Scale bar = 500 μm. b-d: The osteogenesis, adipogenesis, and chondrogenesis differentiation of hBMSC respectively. Scale bar = 200 μm, 100 μm, and 200 μm repectively. e: The surface markers CD105, CD90, CD73, CD45, CD34 of hBMSC by flow cytometry. Figure S2. The verification of STZ-treated diabetic rat model. a The FBG of Control group and STZ group was detected via blood glucose test strips in SD rats on the 0, 5th,10th day. The rats without STZ injection was utilized as Control (n = 3, *p < 0.05). b The body weight of Control group and STZ group was measured by electronic weighing scale (n = 3, *p < 0.05). c The water intake and d food intake of Control group and STZ group was measured through electronic weighing scale (n = 3, *p < 0.05). Table S1. The RNA primers applied for qRT-PCR. [file 13287_2020_1756_MOESM1_ESM.docx]

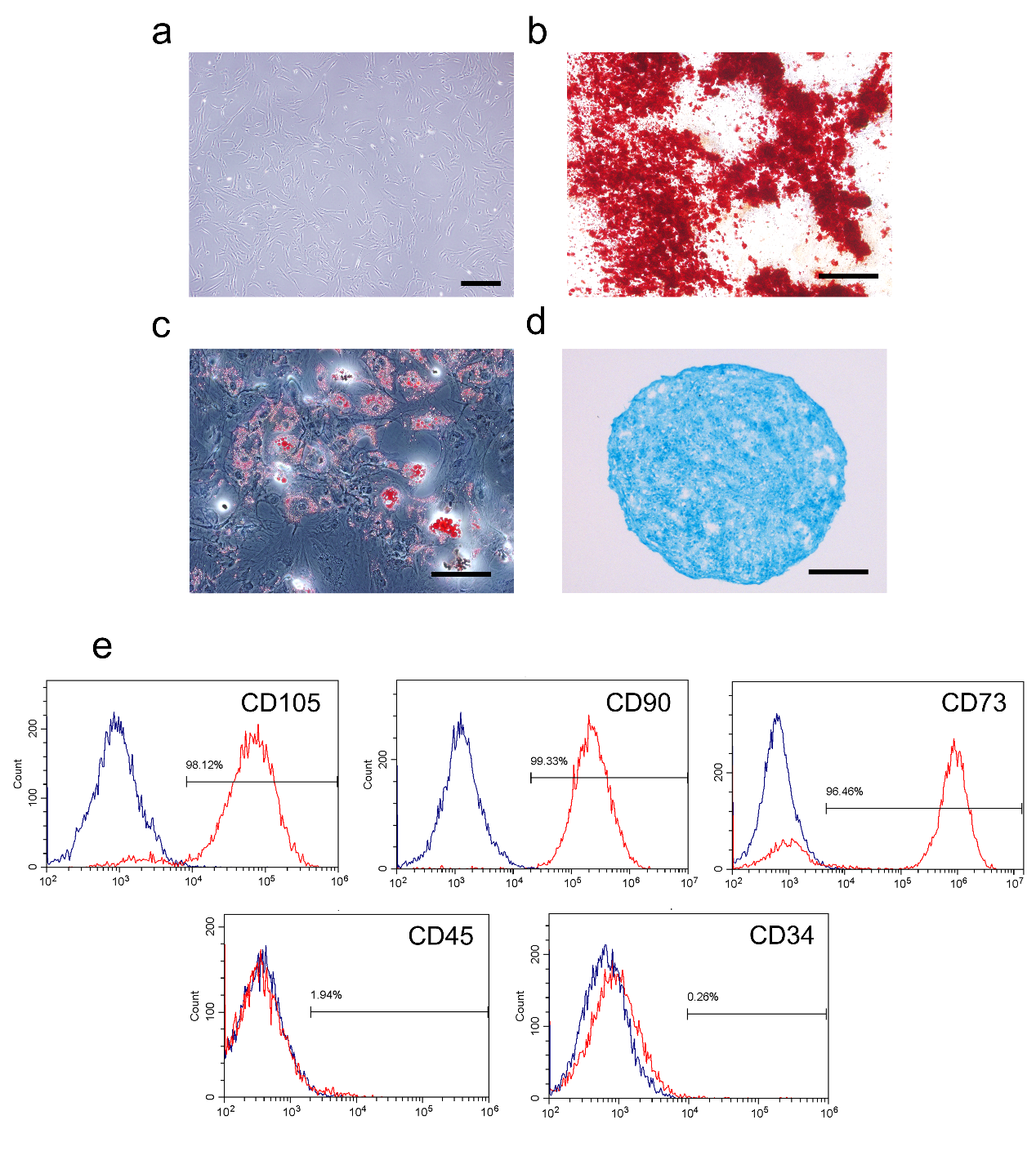


**Fig.S1. The identification of hBMSCs. a**: hBMSCs adhered to plastic culture disk. Scale bar = 500 μm. **b-d**: The osteogenesis, adipogenesis, and chondrogenesis differentiation of hBMSC respectively. Scale bar = 200 μm, 100 μm, and 200 μm repectively. **e**: The surface markers CD105, CD90, CD73, CD45, CD34 of hBMSC by flow cytometry.

**
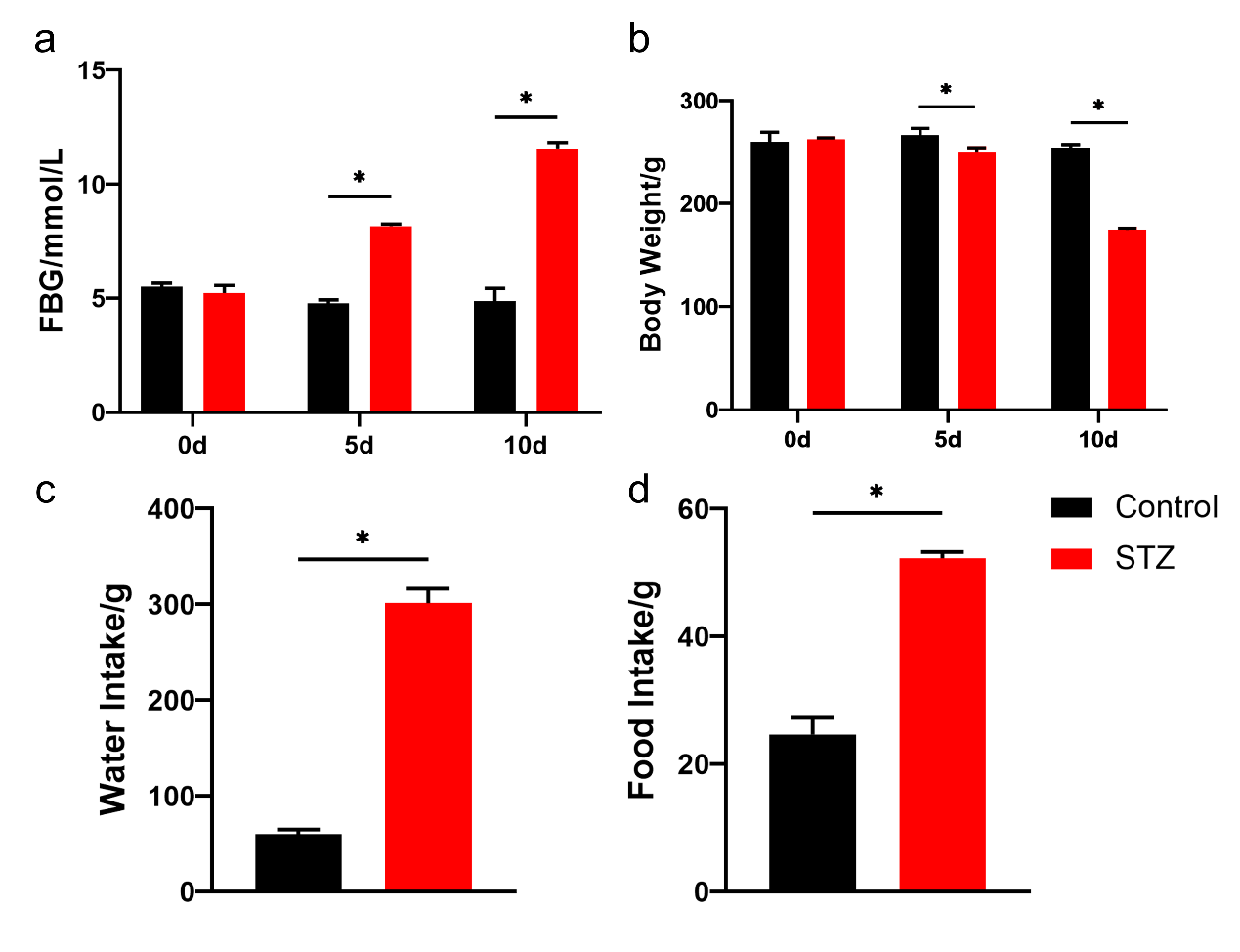
**

**Fig.S2. The verification of STZ-treated diabetic rat model. a** The FBG of Control group and STZ group was detected via blood glucose test strips in SD rats on the 0, 5^th^,10^th^ day. The rats without STZ injection was utilized as control (n=3, *p<0.05). **b** The body weight of Control group and STZ group was measured by electronic weighing scale (n=3, *p<0.05). **c** The water intake and **d** food intake of Control group and STZ group was measured through electronic weighing scale (n=3, *p<0.05).

| Gene | Primers (F=forwards; R=reverse) |
| --- | --- |
| *IL-1β* | F: 5'-TGCAGAGTTCCCCAACTGGTACA-3' |
|  | R: 5'-GTGCTGCCTAATGTCCCCTT-G-3' |
| *TNF-α* | F: 5'-TCAGCCTCTTCTCATTCCTG-3' |
|  | R: 5'-TGAAGAGAAC-CTGGGAGTAG-3' |
| *IL-10* | F: 5'- GCTCT TACTGACTGGCATGAG-3' |
|  | R: 5'-CGCAGCTCTAGGAGCA TGTG-3' |
| *Arg-1* | F: 5'-AACACTCCCCTGACAACCA-3' |
|  | R: 5'-CATCACCTTGCCAATCCC-3' |
| *iNOS* | F: 5'-ATGTCCGAAGCAAACATCAC-3' |
|  | R: 5'-TAATGTCCAGGAAGTAGGTG-3' |
| *18S* | F: 5'-GGACAGGATTGACAGATTGATAG-3' |
| *α-SMA*  *CollagenⅠ*  *Collagen Ⅲ* | R: 5'-CTCGTTCGTTTATCGGAATTAAC-3'  F: 5'-GAGCGTGGCTATTCCTTCGTG-3'  R: 5'-CAGTGGCCATCTCATTTTCAAAGT-3'  F: 5'-CATGAGCCGAAGCTAACCC-3'  R: 5'-CTCCTATGACTTCTGCGTCTGG-3'  F: 5'-CACCCCTCTCTTATTTTGGCAC-3'  R: 5'-AGACTCATAGGACTGACCAAGGTAGTT-3' |

**Table.S1. The RNA primers applied for qRT-PCR.**
